# Ovarian Cancer-Related Hypophosphatemic Osteomalacia—A Case Report

**DOI:** 10.1210/jc.2014-2120

**Published:** 2014-09-02

**Authors:** Hung-An Lin, Shyang-Rong Shih, Yu-Ting Tseng, Chi-Hau Chen, Wei-Yih Chiu, Chih-Yao Hsu, Keh-Sung Tsai

**Affiliations:** Lo-Sheng Sanatorium and Hospital (H.-A.L.), Ministry of Health and Welfare, New Taipei City 242, Taiwan; National Taiwan University College of Medicine (S.-R.S., K.-S.T.), Taipei 100, Taiwan; and Departments of Internal Medicine (S.-R.S., Y.-T.T., W.-Y.C., C.-Y.H., K.-S.T.) and Obstetrics and Gynecology (C.-H.C.), National Taiwan University Hospital, Taipei 100, Taiwan

## Abstract

**Context:**

Tumor-induced osteomalacia (TIO) is a rare paraneoplastic syndrome caused primarily by benign mesenchymal tumors. It has been associated with malignancies in rare cases. High serum levels of fibroblast growth factor (FGF) 23 reported in a group of patients with ovarian cancer had normal serum phosphate levels. There had been no ovarian cancer-related hypophosphatemic osteomalacia in a search of the literature.

**Objective:**

We investigated a 57-year-old woman with progressive low back pain.

**Design and Intervention:**

Clinical, biochemical, and radiological assessments were performed. The patient's serum phosphate and FGF23 levels were evaluated at baseline and after treatment for ovarian cancer.

**Results:**

The patient presented with progressive low back pain and weight loss during the previous 6 months. Imaging studies revealed low bone mineral density and multiple suspicious spinal metastatic lesions. Laboratory examination showed hypophosphatemia, hyperphosphaturia, normocalcemia, an elevated serum alkaline phosphatase level, and an elevated serum FGF23 level. Because TIO was suspected, a tumor survey was performed, and ovarian carcinoma with multiple metastasis was detected. After surgery and chemotherapy treatments for ovarian cancer, the serum phosphate and FGF23 levels returned to normal, and the low back pain improved.

**Conclusions:**

To our knowledge, this is the first case of ovarian cancer-related hypophosphatemic osteomalacia reported in the literature. TIO should be considered in patients with ovarian cancer presenting with weakness, bone pain, and fractures. Investigation of TIO is appropriate when these patients present hypophosphatemia.

Tumor-induced osteomalacia (TIO), one of the causes of hypophosphatemia, is commonly associated with benign mesenchymal tumors of the soft tissue and skeleton (1). Clinical characteristics include renal phosphate wasting, low or normal serum 1,25-dihydroxyvitamin D levels, bone pain, and elevated alkaline phosphatase levels (1). Fibroblast growth factor (FGF) 23, a phosphatonin secreted by these tumors, is responsible for the pathogenesis of TIO (1). Other phosphatonins such as matrix extracellular phosphoglycoprotein, secreted frizzled related protein-4, and FGF7 were also identified as contributing to the pathogenesis of TIO (2). TIO is also associated with malignancies such as prostate cancer, oat cell cancer, hematological malignancies, and colon cancer. In these cases, the primary disease is usually obvious, and treatment is focused on the underlying disease (3, 4). In this study, we report a case of ovarian cancer-related hypophosphatemic osteomalacia, which has not been previously reported in the literature to our knowledge.

## Patient and Methods

### Case description

The 57-year-old woman examined in this study was otherwise healthy before presentation. The patient's menstruation was normal before the onset of menopause at age 52. She had experienced low back pain for 6 months before she visited the outpatient clinic at National Taiwan University Hospital. She also complained of night sweats and a weight loss of 14 kg during the previous 6 months. The low back pain developed while lying down and radiated to both lower limbs. The patient had no abdominal pain, diarrhea, or abnormal vaginal discharge.

### Methods

This study was approved by the Institutional Review Board of the National Taiwan University Hospital (protocol no. 201105045RC) and is registered on Clinicaltrials.gov (protocol no. NCT01660308).

Clinical, biochemical, and radiological assessments were undertaken. The patient's serum phosphate and FGF23 levels were evaluated at baseline and after treatment for ovarian cancer. FGF23 levels were measured using ELISA (Kainos Laboratories, Inc), according to manufacturer's instructions. Two specific murine monoclonal antibodies were bound to the full length of FGF23. One antibody was conjugated to horseradish peroxidase to allow for detection by a spectrophotometric reader. The other antibody was immobilized onto the microtiter well for capture. The normal range for serum FGF23 is 8.2–54.3 pg/mL (5).

## Results

### Physical examination

The patient's height was 155 cm, and her weight was 40 kg (body mass index, 16.6 kg/m^2^). Her conjunctivae were pale. Two firm mass lesions, approximately 2 cm in diameter, were located in the parietal area on both sides. A thyroid nodule, approximately 1 cm in diameter, was noted on palpation. There was no abdominal tenderness or rebound tenderness. Neurological examination revealed normal muscle power and deep tendon reflex of the four limbs.

### Biochemical and imaging studies

Spine radiography revealed a relatively radiolucent texture, scoliosis, and facet arthroses at the lower lumbar spine. With regard to bone mineral density, the lumbar spine had a T-score of −2.42 SD. Magnetic resonance imaging showed multiple spinal metastasis and stenosis over the lumbar spine. Radiography of the skull and extremities showed no obvious osteolytic or osteoblastic lesions. For evaluation of the abnormal bone mineral density and bony lesions, biochemical studies were performed and showed hypophosphatemia (1.6 mg/dL; normal range, 2.7–4.5 mg/dL), normocalcemia (2.2 mmol/L; normal range, 2.02–2.60 mmol/L), an elevated alkaline phosphatase level (597 U/L; normal range, 60–220 U/L), and a normal intact PTH level (17.1 pg/mL; normal range, 16–87 pg/mL). Renal function, serum uric acid level, and liver function were within normal limits (creatinine, 0.7 mg/dL; uric acid, 5.5 mg/dL; aspartate aminotransferase, 26 U/L; alanine aminotransferase, 29 U/L). Hemogram showed elevated white blood cell count (12.66 × 10^3^/μL; normal range, 4–10 × 10^3^/μL) and anemia (red blood cell count, 3.92 × 10^6^/μL; normal range, 3.5–4.5 × 10^6^/μL; hemoglobin, 8.4 g/dL; normal range, 12–15 g/dL). The phosphorus tubular reabsorption rate was lower than normal (75%; normal range, 80–90%). Hyperphosphaturic hypophosphatemia was pronounced, and TIO was highly suspected.

**Figure 1.**
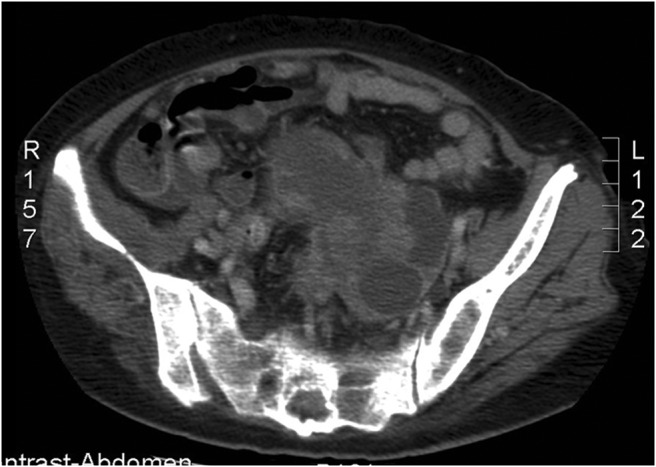
CT scan showing multilobulated cystic lesions over the left adnexa.

**Figure 2.**
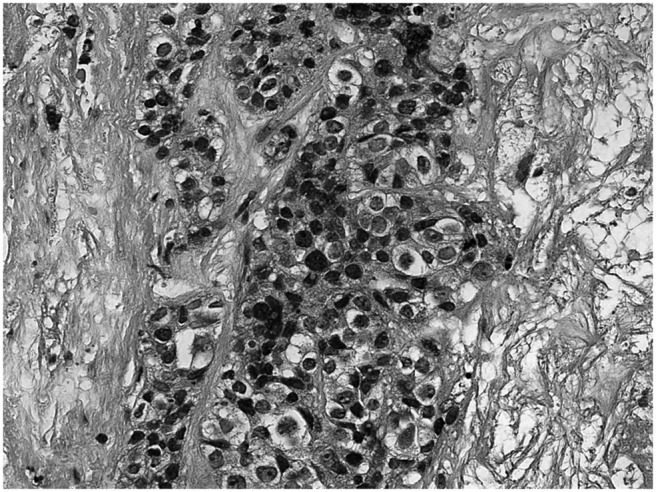
Pathological specimen of the hepatic tumor showing an undifferentiated carcinoma with high cellularity and an infiltrative pattern (magnification, ×400; hematoxylin and eosin stain).

**Figure 3.**
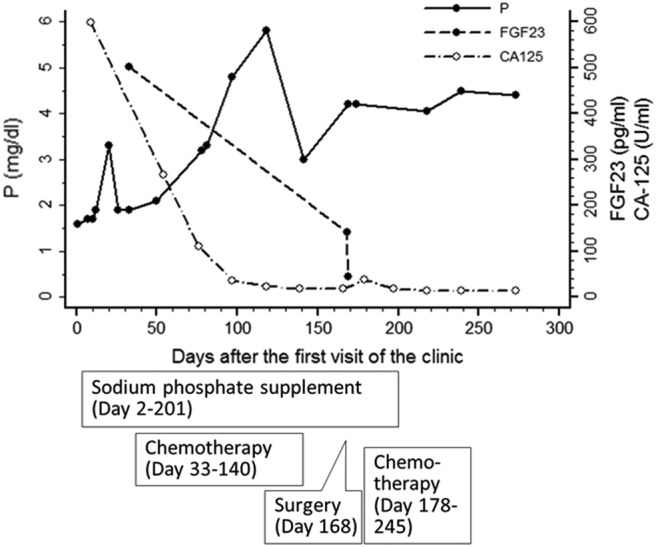
The treatment course and laboratory data. P, Serum phosphate level; CA-125, cancer antigen 125.

The tumor survey revealed a markedly elevated cancer antigen 125 level (598 U/mL; normal range, below 35 U/mL). Abdominal ultrasonography showed multiple tumors in the liver and left adnexa. Computed tomography (CT) showed multiple tumors in the thyroid, bone, lung, and liver, and a large tumor over the uterus and left adnexa (Figure 1). Fine-needle aspiration cytology of the thyroid nodule indicated a poorly differentiated carcinoma. CT-guided biopsy of the hepatic tumor revealed an undifferentiated carcinoma (Figure 2) with strongly positive immunohistochemical staining for cytokeratin 7, p16, Wilms' tumor 1, and β-catenin and negative staining for leukocyte common antigen. Under the diagnosis of stage IV ovarian carcinoma, neoadjuvant chemotherapy with carboplatin and paclitaxel was initiated, followed by debulking surgery including total hysterectomy, bilateral salpingo-oophorectomy, infracolic omentectomy, appendectomy, and pelvic lymph node dissection and adjuvant chemotherapy. The pathological diagnosis was also high-grade serous ovarian carcinoma. The serum FGF23 level decreased gradually after chemotherapy and debulking surgery (initial, 501.6 pg/mL; after neoadjuvant chemotherapy, 140.6 pg/mL; after surgery, 44.141 pg/mL; normal range, 8.2–54.3 pg/mL). The patient's requirement of a phosphate supplement decreased gradually until it was no longer needed after the treatment of ovarian cancer. The treatment course and the changes of laboratory data were presented in Figure 3.

## Discussion

**Figure 4.**
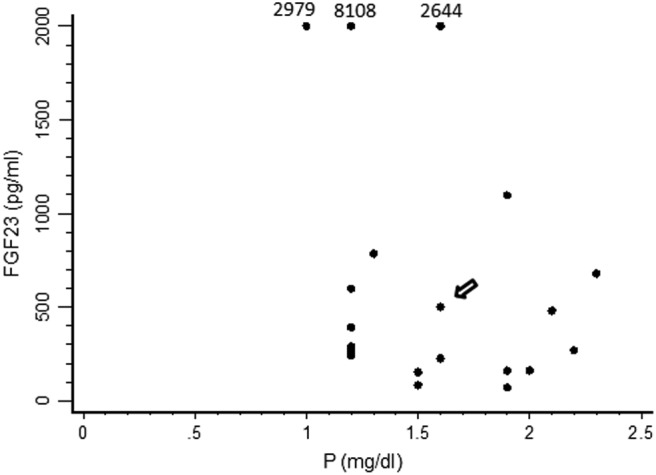
Comparison of serum FGF23 and phosphorus (P) levels between the present case and 19 TIO cases reported in three case series (1, 9, 10). (The arrow points to the present case.) FGF23 levels > 2000 pg/mL are presented at the level of 2000, with the exact data shown above.

We reported a case of high-grade ovarian carcinoma associated with an elevated serum FGF23 level and hyperphosphaturic hypophosphatemic osteomalacia, which has not been previously reported in the literature to our knowledge. TIO is a rare, paraneoplastic syndrome caused primarily by benign mesenchymal tumors. TIO has been associated with malignancies in rare cases (3). Phosphatonins, a group of endocrine hormones, decrease renal tubular reabsorption of phosphate and inhibit 1α-hydroxylation of vitamin D (2). Consequently, muscle weakness, osteomalacia, and even bone fracture may occur (2). The occult course often delays the diagnosis of TIO by an average of 5 years (6). FGF23 helps to diagnose TIO (6). Detailed physical examination, CT, magnetic resonance imaging, octreotide scintigraphy, and positron emission tomography may help to identify tumors (7). Venous sampling of FGF23 has been used to confirm the causative tumor preoperatively (8). Surgical resection of the tumor is the definitive treatment method. Serum FGF23 and phosphate levels typically return to normal within 5 days of the operation (3, 9). After searching the literature, we found neither a case of ovarian cancer-related osteomalacia nor detailed phosphate and FGF23 data of patients with ovarian cancer to draw a figure comparing serum phosphate and FGF23 levels purely in patients with ovarian cancer. Therefore, we compared serum FGF23 and phosphate levels between the present case and 19 other reported cases from three case series (1, 9, 10), in which the location of the responsible tumors included lower and upper extremities, the head, and the spine (Figure 4). We found that FGF23 levels were widely diverse and serum phosphate levels cannot predict FGF23 levels in TIO patients. This is compatible with the conclusion of another study that there were no correlations between FGF23 and the severity of X-link hypophosphatemia, including serum phosphate levels (11).

Ovarian cancer is the leading cause of death due to gynecological malignancies (12). The prognosis is poor, with a 5-year survival rate of approximately 45.6% (13). The most common extra-abdominal metastasis is the pleural space. Other distant metastatic sites include liver, lung, pericardium, bone, and brain. In our patient, the metastatic site involved the thyroid gland, which has been rather uncommon and only occurred in 3–15% of the patients (14, 15). Angiogenesis is responsible for tumor spread and metastasis (16). Several growth factors have been identified to play key roles in driving angiogenesis, such as vascular endothelial growth factor, platelet-derived growth factor, FGF, and the angiopoietin/Tie2 receptor complex (16). FGF is expressed in epithelial ovarian cancer and has proangiogenic properties (13). It directly stimulates the proliferation and migration of endothelial cells, sensitizes epithelial cells to other angiogenic factors, facilitates tube formation, and stimulates the secretion of extracellular matrix remodeling proteases (17, 18). FGF is also secreted into malignant ascites together with vascular endothelial growth factor, which further contributes to ovarian cancer progression and angiogenesis (16). The FGF signaling pathway involves MAPK proteins, proteins of the phosphatidylinositol-3-kinase/Akt cascade, and proteins of the phospholipase-C and inositol triphosphate cascades and may cross talk with other pathways such as the Notch pathway (19, 20). There are 23 FGF isoforms and five receptor molecules identified so far (16). Several isoforms of FGF have been extensively studied in ovarian cancers. For example, serum FGF2 levels were higher in patients with ovarian cancer than in people with benign ovarian tumors or normal ovaries (21); the growth of ovarian cancer was regulated by FGF8 (22); overexpression of FGF18 was an independent predictive marker for poor outcomes in patients with high-grade serous ovarian cancer (23). In a study of 13 women with advanced-stage ovarian cancer, 14 with early-stage ovarian cancer, 14 with benign ovarian tumors, and 39 healthy women, serum FGF23 levels were higher in patients with advanced-stage ovarian cancer, without decreased serum phosphate levels (24). Approximately half of the patients with advanced-stage ovarian cancer had elevated serum FGF23 levels before treatment. This suggests that elevated FGF23 levels in patients with ovarian tumors indicate advanced-stage disease (24). Many women still require surgery to differentiate benign ovarian tumor from cancer because current biochemical markers and imaging techniques are inadequate. Serum FGF23 levels may be a potential candidate for predicting the presence of malignant disease or clinical outcomes, and this is worthy of further study (24). As to the absence of hypophosphatemia in patients with advanced-stage ovarian cancer and high FGF23 in the study mentioned above, one possible explanation is that perhaps other factors are necessary to induce phosphate wasting, such as the overexpression of secreted frizzled related protein-4 and matrix extracellular phosphoglycoprotein (24). Besides, less than five patients with advanced-stage ovarian cancer had FGF23 levels above 500 pg/mL in the study mentioned above (24). The FGF23 level of the patient in our current study was 501.6 pg/mL. The severity of FGF23 level abnormality potentially could contribute to the development of hypophosphatemia to some extent. Other factors that may affect phosphate level are poor appetite and cachexia. Inadequate phosphate intake should also be taken into consideration as one of the reasons for hypophosphatemia in our patient. We reviewed the literature and found no data on the prevalence of hypophosphatemia in cases with ovarian cancer. Hypophosphatemia is associated with weakness, bone pain, and fracture, which may be underdiagnosed as purely cancer cachexia and bone metastasis. In such cases, medical therapy with phosphate supplementation and calcitriol or alfacalcidol may improve weakness and avoid fracture. It should be offered to these patients as in other TIO patients with tumor that cannot be localized or is not surgically resectable (3). Clinicians should have vigilance to measure serum phosphorus and/or FGF23 in patients with ovarian cancer, especially those with advanced-stage cancer or those with weakness, bone pain, and fracture.

In conclusion, a subset of ovarian cancer cases may be associated with elevated FGF23 levels and should be taken into account during the differential diagnosis of TIO. In such cases, hypophosphatemia and bone pain can be relieved with the treatment of ovarian cancer. Besides, TIO should be considered in patients with ovarian cancer presenting with weakness, bone pain, and fractures. Investigation of TIO is appropriate when these patients present hypophosphatemia.
